# Kidney injury molecule-1 expression predicts structural damage and outcome in histological acute tubular injury

**DOI:** 10.1080/0886022X.2019.1578234

**Published:** 2019-03-26

**Authors:** Jieru Cai, Xiaoyan Jiao, Weili Luo, Jing Chen, Xunhui Xu, Yi Fang, Xiaoqiang Ding, Xiaofang Yu

**Affiliations:** a Department of Nephrology, Zhongshan Hospital, Shanghai Medical College, Fudan University, Shanghai, China;; b Shanghai Key Laboratory of Kidney and Blood Purification, Shanghai, China;; c Shanghai Medical Center for Kidney, Shanghai, China;; d Shanghai Institute of Kidney and Dialysis, Shanghai, China

**Keywords:** Acute kidney injury, acute tubular injury, kidney injury molecule-1, renal biopsy, prognosis

## Abstract

**Background:** A few studies have shown that urinary kidney injury molecule-1 (uKIM-1) levels are increased in acute kidney injury (AKI); however, the correlation between uKIM-1 and histological tubular injury, which is considered to be the gold standard for evaluating renal damage and predicting prognosis, is still unclear. We performed this study to determine whether the predicted value of uKIM-1 is correlated with renal KIM-1 (tKIM-1) expression and tissue damage in AKI patients.

**Methods:** This retrospective study recruited 14 healthy individuals and 27 biopsy-proven acute tubular injury (ATI) patients. uKIM-1 and plasma KIM-1 (sKIM-1) levels were measured by ELISA, and tKIM-1 expression was evaluated by immunohistochemistry.

**Results:** Elevated levels of urinary, plasma, and renal KIM-1 were found in ATI patients. The uKIM-1 concentration was positively correlated with tKIM-1 expression and reflected the severity of renal histological injury. The outcome of ATI was associated with uKIM-1 expression: the ATI patients with higher uKIM-1 levels had an increased potential for an incomplete recovery of renal function during follow-up. Additionally, the level of KIM-1, regardless of source, was negatively related to the eGFR, and ROC curve analysis revealed that the ROC-AUC was 0.923 (*p* = 0.000) for the diagnosis of ATI based on a combination of high uKIM-1 and sKIM-1 levels.

**Conclusion:** The uKIM-1 level corresponds with the severity of renal histological damage and can be a potential reliable predictor of adverse renal outcomes in ATI patients. Moreover, combining uKIM-1 and sKIM-1 can increase the sensitivity and specificity of the diagnosis of severe ATI.

## Introduction

Acute kidney injury (AKI) is a common clinical syndrome with a poor prognosis [1]. However, the sCr-based AKI definition provides little information on actual renal dysfunction, which prevents the timely estimation of the severity of the renal injury and administration of possible therapeutic agents [2,3]. The development of a more specific biomarker to evaluate the renal injury and predict adverse outcomes is urgently needed.

Kidney injury molecule-1 (KIM-1) is a transmembrane glycoprotein that is localized at very high levels on the apical membrane of proximal tubular epithelial cells in most injured regions of the kidney [4]. After the injury, the ectodomain of KIM-1 is shed in the urine and serves as a sensitive and early diagnostic indicator of proximal tubular injury in rodents and humans [5–7]. Some reports have indicated that a high level of urinary KIM-1 (uKIM-1) could predict poor outcomes [8,9]. Furthermore, several studies have shown that the expression level of KIM-1 in renal tissue (tKIM-1) could predict the prognosis of various chronic kidney diseases, such as IgA nephropathy, and kidney transplants [10–13]. As a result, uKIM-1 and tKIM-1 have been considered potential prognostic factors for AKI.

Acute tubular injury (ATI), a common complication associated with poor prognosis in hospitalized patients, is the term used to designate AKI resulting from damage to the tubules [14]. ATI is a histologically diagnosed condition, and pathology is the gold standard for evaluating renal damage and predicting prognosis [2]. However, patients with advanced AKI are less likely to be biopsied [15,16]. Therefore, few clinical studies have evaluated the association between the predictive value of uKIM-1/sKIM-1/tKIM-1 with respect to the outcome of AKI and pathological changes or the consistency of variation trend among histological damage, uKIM-1, sKIM-1, and tKIM-1.

The aim of this retrospective investigation was to evaluate (1) possible associations between tissue KIM-1 expression and renal pathological injury in ATI, (2) possible associations between tissue KIM-1 expression and urinary KIM-1 levels in ATI, and (3) the value of uKIM-1/sKIM-1/tKIM-1 in predicting renal function one year after biopsy.

## Materials and methods

### Patients

Patients were recruited from Zhongshan Hospital, Fudan University from June 2015 to June 2017. All procedures and the use of tissue were performed according to national ethical guidelines and approved by the Standing Committee for Clinical Studies of Zhongshan Hospital, Fudan University, in adherence to the Declaration of Helsinki. Informed consent was obtained from patients and healthy volunteers.

The enrollment criteria were as follows: (1) age > 18 years; (2) sCr values within the normal range measured at the time closest to admission within the prior year; (3) clinically diagnosed AKI according to the KDIGO guidelines [16]; and (4) biopsy-proven ATI. The exclusion criteria were as follows: (1) history of chronic kidney disease (estimated glomerular filtration rate (eGFR) < 60 mL/min/1.73 m^2^); (2) renal replacement therapy: maintenance dialysis or after renal transplantation; (3) secondary kidney diseases, such as diabetes, systemic lupus erythematosus, or connective tissue diseases; or (4) active infection, cancer, or a follow-up of <12 months.

The retrospective study included 27 patients diagnosed with ATI. Fourteen healthy subjects served as controls and provided urine and blood samples. Three healthy kidney sections from normal nephrectomy samples adjacent to tumors were used as tissue controls.

### Clinical examination and follow-up

The biochemical parameters in urine and blood were measured on the day of the renal biopsy. The eGFR was calculated by using the CKD-EPI formula. Peak sCr was defined as the highest value recorded in the course of the disease.

Seven days, one month, three months and one year after renal biopsy were set as time points for the evaluation of renal function. According to the KDIGO guidelines, at each time point, the patients were divided into two groups based on the level of eGFR: the complete recovery group (eGFR ≥ 60 mL/min/1.73 m^2^) and the incomplete recovery group (eGFR < 60 mL/min/1.73 m^2^) [16].

### Measurement of KIM-1 in urine and plasma

Urine and blood samples were collected at the time of renal biopsy. Samples were centrifuged at 3000 rpm for 10 min, and the supernatant and plasma separated from the samples were frozen at −80 °C until further analysis. The concentrations of uKIM-1 and KIM-1 in plasma (sKIM-1) were measured in duplicate by ELISA using a commercial kit (R&D Systems, Minneapolis, MN, USA) in accordance with the manufacturer’s guidelines. The uKIM-1 or sKIM-1 level was normalized to urinary or plasma creatinine, respectively, for each sample. The normalized data are indicated as urinary KIM-1 concentration/creatinine concentration (ng/mmol).

### Histopathologic diagnosis and analysis

The renal biopsy specimens were cut at a thickness of 2 μm, and the pathology assessment of ATI was performed as described previously with modifications [2,17]. ATI was evaluated through three criteria: apical blebbing, hydropic swelling, and cell sloughing. The ATI was a 2-level ordinal outcome: mild ATI and severe ATI. We assigned a diagnosis of mild ATI if <25% of tubules were affected and severe ATI if ≥25% of tubules were affected.

KIM-1 expression in renal biopsy specimens was demonstrated by immunohistochemistry (IHC) staining using a mouse anti-human monoclonal antibody against KIM-1 (Abcam, Cambridge, MA, USA) according to the manufacturer’s instructions. Unaffected sections of kidneys from patients with renal cell carcinoma were used as the negative control. The KIM-1 expression level was scored semiquantitatively by counting the stained tubules under 400× magnification as described previously with modifications [10]: 0 tubules indicated grade 0; 1–5 tubules, grade 1; 6–10 tubules, grade 2; and more than 10 tubules, grade 3.

### Statistical analysis

The data are presented as the means ± SD. Differences in quantitative parameters with normal distribution between groups were assessed using a *t* test. Differences in quantitative parameters with nonnormal distribution and qualitative data between groups were assessed using a nonparametric test. Spearman and partial correlation analyses were used to analyze correlations among various parameters. The sensitivity and specificity of uKIM-1/uCr, sKIM-1/sCr, and tKIM-1 as markers of the severity of renal damage (severe ATI) were compared using ROC curves. A *p* value less than 0.05 was considered to indicate statistical significance. The analyses were performed with SPSS statistical software package (version 19.0, IBM, NY, USA).

## Results

### Clinical characteristics of control and ATI patients

In the retrospective study, 27 ATI patients were enrolled, and 14 healthy subjects served as controls for the urine and blood parameter assessments. The clinical characteristics of patients and healthy subjects are shown in Table 1. There were no significant differences between the two groups in these clinical data.

**Table 1.** Clinical characteristics of the study subjects.

Characteristic	Normal (*N* = 14)	ATI (*N* = 27)	*p* Value
Age, years	42.86 ± 12.79	48.15 ± 15.33	0.362
Gender			
Male (n, %)	9 (64.3%)	21 (77.8%)	0.355
Female	5 (35.7%)	6 (22.2%)	
ALT (IU/L)	17.21 ± 7.47	18.41 ± 11.22	0.934
AST (IU/L)	22.93 ± 5.64	27.94 ± 7.19	0.179
Albumin (g/L)	38.36 ± 3.39	36.59 ± 8.21	0.967
Baseline sCr (μmol/L)	53.75 ± 10.71	61.02 ± 15.79	0.173

The data were presented means ± SD. AKI: acute kidney injury; ALT: alanine aminotransferase; AST: aspartate aminotransferase; sCr: serum creatinine.

Based on the pathology assessment of ATI, 13 of the 27 ATI patients were in the mild ATI group, and 14 patients were in the severe ATI group. The parameters of renal function are listed in Table 2. Patients in the severe ATI group had higher urinary albumin-to-creatinine ratio (ACR) than those in the mild ATI group (201.10 ± 84.68 mg/mmol vs. 92.73 ± 74.04 mg/mmol, *p* = 0.007). eGFR was significantly lower in the severe ATI group than in the mild ATI group (23.07 ± 21.66 mL/min vs. 40.54 ± 21.09 mL/min, *p* = 0.020). No significant differences in baseline sCr, Peak sCr, 24 h proteinuria, blood urea nitrogen (BUN), or sCr on the day of biopsy were detected between the two groups.

**Table 2.** Clinical characteristics of ATI patients.

Characteristic	Mild ATI (*N* = 13)	Severe ATI (*N* = 14)	*p* Value
Baseline sCr (μmol/L)	65.41 ± 17.71	56.94 ± 13.13	0.174
ACR (mg/mmol)	92.73 ± 72.04	201.10 ± 84.68	0.007**
24h Proteinuria (g/d)	0.31 ± 0.23	0.41 ± 0.16	0.132
Peak sCr (μmol/L)	332.40 ± 120.90	489.60 ± 320.96	0.520
Renal biopsy			
sCr (μmol/L)	218.00 ± 106.34	405.90 ± 298.80	0.114
BUN (μmol/L)	9.59 ± 3.95	13.56 ± 8.57	0.732
eGFR(ml/min)	40.54 ± 21.09	23.07 ± 21.66	0.020*

The data were presented means ± SD. ATI: acute tubular injury; sCr: serum creatinine; ACR: urinary albumin to creatinine ratio; BUN: blood urea nitrogen; eGFR: estimated glomerular filtration rate.

*p < 0.05.

**p < 0.01.

### The relationship between KIM-1 level in renal tissue and kidney tissue damage

**Figure 1.**
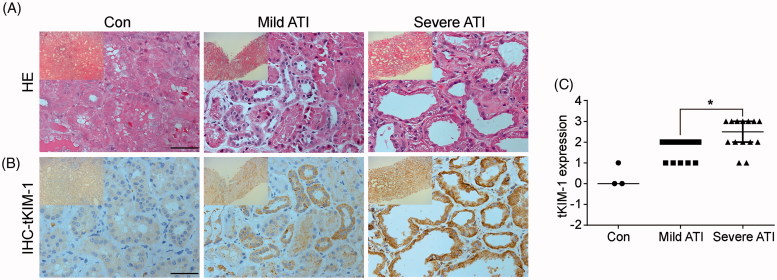
KIM-1 levels in renal tissue were correlated with kidney tissue damage. (A) Representative images of HE-stained renal biopsy specimens, scale bar: 50 µm. (B) Representative immunohistochemical staining image of KIM-1 expression in renal tissue, scale bar: 50 µm. (C) Semiquantitative analysis of the tKIM-1 expression level in control and ATI patients. Data are expressed as the means ± SD. *p < 0.05.

The control tissues were acquired from the normal nephrectomy samples adjacent to tumors, and the damaged tissues in the ATI groups were obtained from the renal biopsies. The severe ATI group showed more severe tubular damage than did the mild ATI group (Figure 1(A)). The expression of KIM-1 in renal tissue was detected by IHC. The expression of tKIM-1 was almost undetectable in the control group, while the expression of tKIM-1 was increased in the ATI group, especially in the severe group (1.46 ± 0.66 vs. 2.21 ± 0.70, *p* = 0.012; Figure 1(B,C)). A chi-square test indicated that tKIM-1 was positively correlated with kidney tissue damage (*p* = 0.045). Therefore, the level of tKIM-1 depended on the extent of tissue damage: tKIM-1 increased significantly as the renal damage score increased.

### Urinary and plasma KIM-1 detection by ELISA

**Figure 2.**
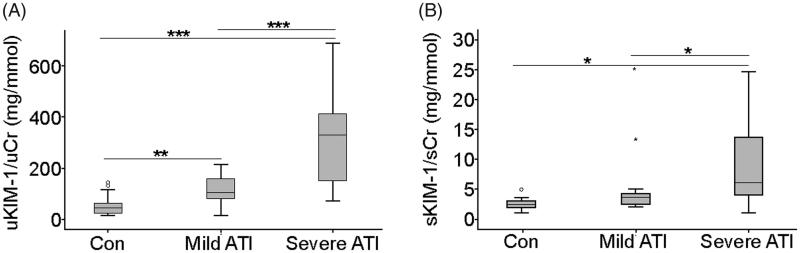
Urinary and plasma KIM-1 concentrations in healthy controls and ATI patients. (A) uKIM-1/uCr levels in healthy controls and ATI patients. (B) sKIM-1/sCr levels in healthy controls and ATI patients. Data are expressed as the means ± SD. *p < 0.05; **p < 0.01; ***p < 0.001.

The expression of uKIM-1/uCr was at very low levels in healthy controls (57.99 ± 43.07 mg/mmol), but the expression increased dramatically after injury in ATI patients. A significant difference was observed in the ATI subgroup, and the severe ATI group showed a higher uKIM-1/uCr concentration than did the mild ATI group (316.18 ± 163.80 mg/mmol vs. 117.61 ± 62.61 mg/mmol, *p* = 0.001; Figure 2(A)). The above trend was also found for sKIM-1/sCr. The plasma level of KIM-1 was low in the healthy control group, higher in the mild ATI group, and highest in the severe ATI group (Figure 2(B)).

### Relationship among tKIM-1, uKIM-1 and sKIM-1 and predictive value for severe ATI

**Figure 3.**
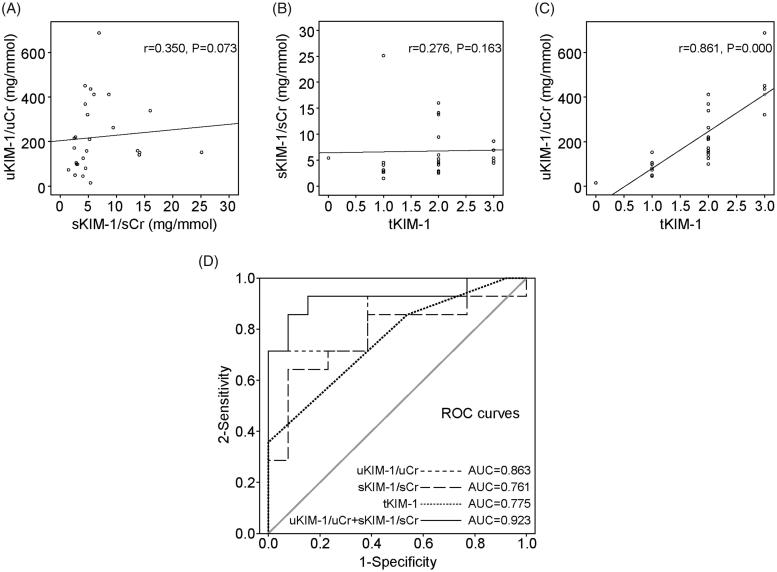
Correlation of KIM-1 with clinical parameters of renal function in ATI patients. (A) The correlation between uKIM-1/uCr and sKIM-1/sCr levels. (B) The correlation between tKIM-1 and sKIM-1/sCr levels. (C) The correlation between tKIM-1 and uKIM-1/uCr levels. (D) ROC curves to evaluate the sensitivity and specificity for severe ATI.

There was a significant correlation between uKIM-1/uCr and tKIM-1 levels (*r* = 0.861, *p* = 0.000), whereas there was no significant correlation between uKIM-1/uCr and sKIM-1/sCr levels or tKIM-1 and sKIM-1/sCr levels (Figure 3(A–C)).

Because severe ATI more likely resulted in a reversible decrease in renal function and increased the risk of death, ROC curve analysis was performed to test the sensitivity and specificity of KIM-1 as a marker of the severity of renal damage (Figure 3(D)). The area under the ROC curve (ROC-AUC) values for predicting the severity of renal damage with uKIM-1/uCr, sKIM-1/sCr, and tKIM-1 were 0.863, 0.761, and 0.775, respectively. Notably, the ROC-AUC value for the diagnosis of severe ATI significantly increased, up to 0.923, on the basis of combining uKIM-1/uCr with sKIM-1/sCr. The sensitivity was 84.6%, and the specificity was 14.3% when the cutoff point for the uKIM-1/uCr level was 158.5 mg/mmol ng/mg and when that for the sKIM-1/sCr level was 4.61 mg/mmol.

### Correlation of KIM-1 with clinical parameters of renal function in ATI patients

To identify the association between clinical variables of renal functions with higher levels of urinary, renal or plasma KIM-1, we performed correlation analysis between these clinical variables and KIM-1 at the time of renal biopsy in all ATI patients. As shown in Table 3, uKIM-1/uCr was positively correlated with 24 h proteinuria (*r* = 0.456, *p* = 0.038), ACR (*r* = 0.525, *p* = 0.031), and sCr (*r* = 0.558, *p* = 0.006) and was negatively correlated with eGFR (*r* = 0.6525, *p* = 0.000). However, uKIM-1/uCr was not correlated with peak sCr or BUN. Furthermore, sKIM-1/sCr levels were negatively correlated with BUN (*r* = 0.705, *p* = 0.001) and eGFR (*r* = 0.719, *p* = 0.001). There was no correlation between sKIM-1/sCr and 24 h proteinuria, ACR, peak sCr or sCr. In addition, tKIM-1 expression levels were positively correlated with sCr (*r* = 0.499, *p* = 0.011) and negatively correlated with eGFR (*r* = 0.546, *p* = 0.003).

**Table 3.** Correlation of urinary, plasma and renal KIM-1 express levels and clinical indexes in ATI patients.

Variants	uKIM-1/uCr		sKIM-1/sCr		tKIM-1	
	*r*	*p*	*r*	*p*	*r*	*p*
24h Proteinuria	0.456	0.038*	0.134	0.563	0.408	0.066
ACR	0.525	0.031*	0.401	0.124	0.481	0.051
Peak sCr	0.299	0.187	−0.373	0.096	0.293	0.197
Renal biopsy						
eGFR	−0.652	0.000***	−0.719	0.001**	−0.546	0.003**
BUN	0.356	0.124	−0.705	0.001**	0.352	0.128
sCr	0.558	0.006**	0.047	0.824	0.499	0.011*

KIM-1: kidney molecule injury-1; ATI: acute tubular injury; uKIM-1/uCr: urinary KIM-1/urinary creatinine; sKIM-1/sCr: plasma KIM-1/plasma creatinine; tKIM-1: renal tissue KIM-1; ACR: urinary albumin to creatinine ratio; sCr: plasma creatinine; BUN: blood urea nitrogen; eGFR: estimated glomerular filtration rate.

*p < 0.05.

**p < 0.01.

***p < 0.001.

### Analysis of prognosis predictions on the basis of KIM-1 levels

Outcomes were evaluated at the following time points: seven days, one month, three months and one year after renal biopsy. As shown in Table 4, the uKIM/uCr level in the incomplete group was higher than that in the complete group at each follow-up time point, though a significant difference was noted only at the seven-day follow-up time point (259.42 ± 124.03 mg/mmol vs. 154.54 ± 197.17 mg/mmol, *p* = 0.010). Similar trends were detected for sKIM-1/sCr and renal KIM-1 expression. In short, a poor prognosis of ATI was associated with a high KIM-1 level, regardless of source, and patients with a higher KIM-1 level had an increased potential for an incomplete recovery of renal function during follow-up.

**Table 4.** The relationship between KIM-1 and recovery of renal function in ATI patients during the follow-up time.

Follow-up time	uKIM-1/uCr			sKIM-1/sCr			tKIM-1		
	Complete recovery	Incomplete recovery	*p* Value	Complete recovery	Incomplete recovery	*p* Value	Complete recovery	Incomplete recovery	*p* Value
7 days	154.54 ± 197.17 (*n* = 10)	259.42.54 ± 124.03 (*n* = 17)	0.010*	4.57 ± 3.60 (*n* = 10)	8.02 ± 6.04 (*n* = 17)	0.035*	1.40 ± 0.84 (*n* = 10)	2.11 ± 0.60 (*n* = 17)	0.020*
1 month	194.94 ± 168.35 (*n* = 19)	281.46 ± 125.04 (*n* = 8)	0.071	5.50 ± 3.62 (*n* = 19)	9.70 ± 7.92 (*n* = 8)	0.152	1.79 ± 0.85 (*n* = 19)	2.00 ± 0.53 (*n* = 8)	0.523
3 months	201.12 ± 163.24 (*n* = 24)	336.73 ± 115.46 (*n* = 3)	0.066	5.88 ± 3.83 (*n* = 24)	7.73 ± 7.22 (*n* = 3)	0.867	1.81 ± 0.80 (*n* = 24)	2.33 ± 0.58 (*n* = 3)	0.273
1 year	201.12 ± 163.24 (*n* = 24)	336.73 ± 115.46 (*n* = 3)	0.066	5.88 ± 3.83 (*n* = 24)	7.73 ± 7.22 (*n* = 3)	0.867	1.81 ± 0.80 (*n* = 24)	2.33 ± 0.58 (*n* = 3)	0.273

KIM-1: kidney molecule injury-1; ATI: acute tubular injury; uKIM-1/uCr: urinary KIM-1/urinary creatinine; sKIM-1/sCr: plasma KIM-1/plasma creatinine; tKIM-1: renal tissue KIM-1.

*p < 0.05.

## Discussion

In this study, we found elevated levels of urinary, plasma and renal KIM-1 in ATI patients. The uKIM-1 concentration was positively correlated with tKIM-1 expression, reflecting the severity of renal histological injury. The outcome of ATI was associated with KIM-1 expression: the ATI patients with higher KIM-1 levels had an increased potential for an incomplete recovery of renal function during follow-up. Additionally, the level of KIM-1 was negatively related to eGFR, and ROC curve analysis revealed that the ROC-AUC was 0.923 (*p* = 0.000) for the diagnosis of ATI based on a combination of high uKIM-1 and sKIM-1 levels.

ATI is one of the most common causes of AKI with tubular damage and is associated with some adverse outcomes [18]. Early prognostic predictive biomarkers are essential for guiding disease management and could improve patient outcomes [19]. ATI is a histological diagnosis, and its prognosis is dependent on the severity of tubular damage [2]. However, renal biopsy is an invasive procedure, and patients with advanced AKI are not frequently biopsied [16]. Therefore, it is necessary to find biomarkers that are clearly correlated with histopathologic changes, easily and accurately measurable, and predictive of the outcome of ATI.

A previous study showed that KIM-1 expression in tissue correlated well with tubulointerstitial nephritis features in different chronic kidney diseases, such as IgA nephropathy, kidney transplants, and adult Henoch-Schonlein purpura nephritis [10,11,20]. This result was also confirmed in ATI by this study. Urinary KIM-1 is mainly derived from tissue KIM-1, which is expressed in damaged renal tubular epithelial cells [21,22]. We found a positive correlation between tissue KIM-1 expression and renal urinary KIM-1 levels; therefore, we speculated that urinary KIM-1 concentration could be a specific biomarker to detect renal injury.

As mentioned above, urinary KIM-1 could indirectly reflect the pathological changes in ATI [23]. Does the urinary KIM-1 level predict the outcome in ATI? Recently, an observational study reported that urinary KIM-1 levels were predictive of renal prognosis in patients with AKI, but that study focused only on changes in laboratory parameters of renal function without histological samples [8]. Similarly, this conclusion was confirmed in this study. We considered that the positive relationship between urinary KIM-1 and pathological scores from this study could explain the above statements. Theoretically, renal KIM-1 expression could also be considered a prognostic factor for ATI. Due to the scarcity of pathological specimens, urinary KIM-1 is more suitable for clinical application than tissue KIM-1 expression.

To clarify the predicted value of urinary KIM-1 in ATI, follow-up at different time points was recorded. At each follow-up time, the patients were divided into two groups based on renal function: complete recovery and incomplete recovery. At each follow-up time point, the patients in the complete recovery group presented lower uKIM-1 levels than those of the incomplete recovery group. In other words, a higher urinary KIM-1 level on the day of biopsy, reflecting the histologic damage, had more potential to predict an incomplete recovery of renal function. After a regular one-year follow-up, the number of patients in the incomplete recovery group decreased, and that in the complete recovery group increased. Therefore, high urinary KIM-1 was related to a greater possibility of a poor outcome.

Additionally, we compared the relationships between KIM-1 and eGFR. Regardless of the source of KIM-1, univariate analysis showed that KIM-1 levels were highly correlated with eGFR. To some extent, KIM-1 was a biological marker of ATI. To understand the diagnostic significance of KIM-1 in ATI, ROC curve analysis was performed and revealed that the ROC-AUC was 0.863 (*p* = 0.001) and 0.761 (*p* = 0.021) for the diagnosis of ATI on the basis of uKIM-1 and on the basis of skim-1, respectively. Combining uKIM-1 with sKIM-1 dramatically increased the ROC-AUC to 0.923 (*p* = 0.000), significantly elevating the sensitivity and specificity of the diagnosis of severe ATI. For a patient who is strongly suspected to have ATI but is not suitable enough to undergo renal biopsy, it can be considered to test the level of KIM-1 in urine and plasma to evaluate the severity of renal damage. A high KIM-1 level was valuable in the diagnosis of severe ATI, and active management could improve the clinical prognosis.

This study had several limitations. First, this retrospective study was a small sample size. It was conducted at a single center with strict selective criteria, but it could be the basis for a larger, multicenter study to validate the reliability among the urinary/plasma KIM-1 level, renal KIM-1 expression, and severity of renal damage. Second, the changes in renal function were assessed for only one year, which was a relatively short-term follow-up. Long-term follow-up will be needed to show the value of uKIM-1 concentration or renal KIM-1 expression for monitoring renal prognosis.

## Conclusion

The urinary KIM-1 level is consistent with the severity of renal histological damage and can be a potential reliable predictor of adverse renal outcomes in ATI patients. Moreover, combined uKIM-1 and sKIM-1 can increase the sensitivity and specificity of the diagnosis of severe ATI, which can help the clinician to engage in early interventions, especially for these the patients who are strongly suspected to have ATI but is not suitable enough to perform renal biopsy.
